# Effect of Food Preparations on In Vitro Bioactivities and Chemical Components of *Fucus vesiculosus*

**DOI:** 10.3390/foods9070955

**Published:** 2020-07-18

**Authors:** Rebeca André, Laura Guedes, Ricardo Melo, Lia Ascensão, Rita Pacheco, Pedro D. Vaz, Maria Luísa Serralheiro

**Affiliations:** 1 BioISI—Biosystems & Integrative Sciences Institute, Faculdade de Ciências, Universidade de Lisboa, 1749-016 Lisboa, Portugal; rebeca.esperanca@gmail.com (R.A.); lrguedes@fc.ul.pt (L.G.); rpacheco@deq.isel.ipl.pt (R.P.); 2 Marine and Environmental Sciences Centre (MARE), Faculdade de Ciências, Universidade de Lisboa, 1749-016 Lisboa, Portugal; ramelo@fc.ul.pt; 3 Centre for Environmental and Marine Studies (CESAM) Faculdade de Ciências, Universidade de Lisboa, 1749-016 Lisboa, Portugal; lmpsousa@fc.ul.pt; 4 Department of Chemical Engineering, ISEL—Instituto Superior de Engenharia de Lisboa, Rua Conselheiro Emídio Navarro, 1, 1959-007 Lisboa, Portugal; 5 Champalimaud Foundation, Champalimaud Centre for the Unknown, 1400-038 Lisboa, Portugal; pedro.vaz@fundacaochampalimaud.pt; 6 Department of Chemistry and Biochemistry, Faculdade de Ciências, Universidade de Lisboa, Campo Grande, C8 bldg, 1749-016 Lisboa, Portugal

**Keywords:** *Fucus vesiculosus*, phlorotannins, peptides, acetylcholinesterase, HMG-CoA reductase, cholesterol, LC-HRMS/MS

## Abstract

*Fucus vesiculosus* is a brown macroalgae used in food and generally considered safe to be consumed, according to EU Directive (EC 258/97). The aim of this study is to analyze the effect of food preparation on *F.vesiculosus* of different origins on what concerns its chemical constituents and final bioactivities. The aqueous extract of the seaweeds were obtained at different temperatures, similar to food preparation and then purified by SPE. The compound identification was carried out by Liquid Chromatography High Resolution Mass Spectrometry (LC-HRMS/MS) and algae extracts microstructure were observed by Scanning Electron Microscopy (SEM). The activities were determined by using antioxidant activity, inhibition of acetylcholinesterase (AChE) and 3-hidroxi-3-methyl-glutaril-CoA (HMG-CoA) reductase (HMGR) together with Caco-2 cells line simulating the intestinal barrier. The activity of AChE and the HMGR were inhibited by the extracts giving IC_50_ values of 15.0 ± 0.1 µg/mL and 4.2 ± 0.1 µg/mL, respectively and 45% of the cholesterol permeation inhibition. The main compounds identified were phlorotannins and peptides derivatives. The mode of preparation significantly influenced the final bioactivities. Moreover, the in vitro results suggest that the preparation of *F. vesiculosus* as a soup could have hypercholesterolemia lowering effect.

## 1. Introduction

Macroalgae have been used for centuries as a source of food, fuel, feed, and agricultural fertilizers [1]. The consumption of seaweeds has been growing in the past years because people are looking for a more environmentally friendly and healthier lifestyle, being aware of the influence of diet on health and well-being [2,3]. The global seaweed industry produces approximately 12 million tons per annum in volume of which 85 percent comprises food products [4].

*Fucus vesiculosus* L. (Fucaceae), commonly known as bladderwrack, is a brown macroalgae used as “sea-vegetable” to make tea or in cooked dishes, soups, or sprinkled in salads, despite having a strong iodine and very salty taste [5,6,7]. In traditional medicine this seaweed is administered orally for different conditions such as weight loss, prevention of atherosclerosis, viscous blood and hypercholesterolemia, mineral deficit, complaints, arthritis, arthrosis, and as an adjuvant for menopause [8,9,10,11]. Traditionally it is also administered topically for obesity and arthritis [8]. *F. vesiculosus* grows in different environmental conditions from saline lagoons to rocky shores and it is widespread along the coastlines worldwide [5]. Its chemical composition depends on the harvest season, geographic location, and environmental factor such as substrate firmness, exposure to ice and waves, salinity, wave force, light, or competition between macroalgae [12,13]. However, it is essentially constituted by polyphenolic compounds, proteins, minerals, iodine, vitamins, fatty acids, and non-digestible polysaccharides [7,12].

Phlorotannins, the phenolic compounds high in concentration in brown algae, are highly hydrophilic, have a molecular size from 234 to more than 100,000 Da [14] and are composed of oligomers of phloroglucinol (1,3,5-Trihydroxybenzene) [15]. According to different studies, phlorotannins reaches 15% of *F. vesiculosus* dry weight and are characterized by low toxicity and high antioxidant activity [16,17]. Phlorotannins have also been described as having in vitro anti-cancer, anti-diabetic, anti-lipidemic, and anti-hypertensive activities, among others [6,18,19,20] and therefore have been used as food additives [3].

Recent studies have found that protein hydrolysates from macroalgae extracts contain peptides showing various in vitro activities, such as antimicrobial, antihypertensive, anticoagulant, and anticancer activity. Some studies have also shown the presence of peptides capable of lowering plasma cholesterol levels [18,21] and with antioxidant activity [22]. The bioactive peptides found are usually composed of hydrophobic amino acid residues (aromatic or branched side chain) like histidine, proline, tyrosine, and tryptophan, which were also associated with high antioxidant activity [2].

The development of seaweed-derived ingredients for cosmetic, pharmaceutical, and food industries is a growing research area [23]. Considering that *F. vesiculosus* is an edible alga, it will be studied using different aqueous extracts prepared under distinct conditions similar to food preparations. The antioxidant activity of the extracts is evaluated, and one of their main components, phlorotannins, has been reported as having antioxidant activity [17]. Antioxidants may neutralize the excessive formation of reactive species and free radicals inside the cell, contributing to the prevention of cell damage in different diseases [24]. The capacity of the extracts to inhibit acetylcholinesterase (AChE) (EC 3.1.1.7) enzyme is also studied. The enzyme AChE is located in the synaptic gaps and neuromuscular junctions and its reversible inhibition is used as an approach to treat gastrointestinal disorders and Alzheimer’s disease [25]. Different studies with phlorotannins derivatives have demonstrated that this type of compounds has in vitro inhibitory potential against AChE [26,27].

Also, the capacity of the extracts to reduce hypercholesterolemia, one of the major risks to cardiovascular diseases and the principal cause of mortality in Europe [28], is evaluated by inhibition of 3-Hydroxy-3-methyl-glutaryl-coenzyme A reductase (HMGR) (E.C.1.1.1.34), the enzyme that regulates the biosynthesis of intracellular cholesterol; also the effect on the in vitro cholesterol cell permeability in the intestinal lining is studied. There are still no studies with aqueous extracts of *F. vesiculosus*, but it has already been demonstrated the Seapolynol^™^, a commercially available *Ecklonia cava* extract and the purified phlorotanin dieckol show inhibitory capacity for HMGR in vitro [20].

In addition, the microstructure of the different extracts is observed by scanning electron microscopy (SEM) and a comparative analysis of the Liquid-Chromatography-High Resolution Mass Spectrometry (LC-HRMS/MS) chromatograms of the extracts is done in an attempt to provide a scientific explanation for the different results of the bioactivities found in the *F. vesiculosus* extracts from different origins (ocean, Tagus, and commercial capsules) and under the effect of different food preparations.

The aim of the present study is to evaluate the effect of the seaweed origin and the cooking processes on the final antioxidant activity, digestion improvement, and cholesterol biosynthesis and intestinal permeability, as information on the effect of phlorotannins on these items is scarce. To accomplish these objectives different algae origin, drying processes, and different aqueous extraction methods are performed, and the described bioactivities are evaluated using the extracts obtained.

## 2. Materials and Methods

### 2.1. Chemicals

All chemicals were of analytical grade. Water, methanol (MeOH), formic acid, and acetonitrile LC-MS grade Optima were purchased from Fisher Scientific (Hampton, USA). Roswell Park Memorial Institute (RPMI) medium, HBSS (Hanks balanced salt solution), glutamine, Pen-Strep (penicillin and streptomycin mixture), and FBS (fetal bovine serum) were bought from Lonza (Verviers, Belgium). Tris(hydroxymethyl)aminomethane (Tris), sodium chloride, and sodium carbonate were obtained from Merck kGaA (Darmstadt, Germany). Magnesium chloride hexahydrate was obtained from PanReac (Barcelona, Spain). Acetylcholinesterase (AChE), acetylcholine iodide (AChI), 5-5′-Dithiobis (2-nitrobenzoic acid) (DTNB), HMGR assay kit, 2,2-Diphenyl-1-picrylhydrazyl (DPPH), Folin-Ciocalteu’s phenol reagent, and phloroglucinol ≥99.0% ( HPLC - High Performance Liquid Chromatography) were bought from Sigma-Aldrich (Barcelona, Spain).

### 2.2. Algae Material

Samples of the brown seaweed *F. vesiculosus* Linnaeus from three different sources were studied: (1) Harvested during low tide in the Tagus estuary (Lisbon, 15 February, 2018), named here as Tagus samples; (2) commercially available whole dried algae (imported by Américo Duarte Paixão Lda and commercialized by Celeiro diet™, Lisbon, Portugal, Lot number 03ALG2731901) from the North Atlantic Ocean, denominated here as ocean samples; and (3) capsules sold in health food stores as food supplement (sold by Dalipharma™, Lot number D325), named here as capsule samples, label indicates that the capsules contain midrib of *F.vesiculosus* (83%), tricalcium phosphate, lactose, and magnesium stearate.

Fresh Tagus fronds were washed with distilled water; the fertile receptacle regions were removed, and the fronds were sectioned into small pieces before being lyophilized, and then finely pulverized, and stored at −20 °C until analysis. In parallel, other Tagus fronds were dried also in the oven at 180 °C.

### 2.3. Aqueous Extract Preparation

Aqueous extracts were prepared with two extraction conditions: (1) 24 h of incubation with water at room temperature (25 °C); (2) boiling (decoction) for 30 min.

The extract preparation with water at room temperature was carried out; 5 g of the algae powder was mixed with 100 mL of distilled water, incubated in a platform shaker for 24 h at 200 rpm and at room temperature. Decoction was prepared using 5 g of the algae powder boiled for 30 min in 100 mL of distilled water, similar to a soup preparation. Both extracts were centrifuged at 5000× *g* for 10 min at 4 °C. The supernatants were lyophilized and then stored at −20 °C until analysis. Both extractions conditions were done with two replicates.

### 2.4. SPE Purification

The extracts from Tagus and the oceans samples, extracted as decoctions, were purified as described in [29], with some modifications. The extracts were resuspended with water to a concentration of 30 mg/mL and loaded into a Sep-Pak C18 Plus Short Cartridge (360 mg sorbent per cartridge, 55–105 μm particle size, 50/pk [WAT020515]) (Waters, Milford, MA, USA), which had been pre-conditioned with methanol followed by water, and then washed with water. Phlorotannins were eluted with methanol and the solvent was evaporated under compressed air. The resulting purified fraction rich in phlorotannins was resuspended in water and centrifuged in an Eppendorf 5415D equipment, DE (Marshall Scientific, Hampton, NH, United States) at 3500× *g* for 10 min. This SPE fraction (eluted with methanol) was used for LC-HRMS/MS analysis, and for the biological activities under study. The purification process resulted in a concentration factor of 4.8 and 11.4 for the Tagus and ocean sample, respectively.

### 2.5. Chemical Analysis by HPLC-DAD and LC-HRMS/MS

The high-performance liquid chromatographic (HPLC) analysis was carried out in an Elite LaChrom^®^ VWR Hitachi liquid chromatograph (Tokyo, Japan) equipped with a Column oven L-2300 and Diode array detector L-2455 (VWR, USA). A column LiChroCART^®^ 250-4 LiChrospher^®^ 100 RP-18 (5 μm) was used. The extracts in study were analyzed injecting 25 µL with an auto injector and using a gradient composed of 0.05% trifluoroacetic acid (solution A), and acetonitrile (solution B) as follows: 0 min, 100% A; 30 min, 70% A, 30% B; 40 min, 20% A, 80% B; 45 min, 20% A, 80% B; 50 min 70% A, 30% B; 52 min, 100% A; 55 min 100% A. The flow rate was 0.8 mL/min and the detection were carried out between 200 and 600 nm using a diode-array detector (DAD). The chromatograms were extracted by representing the highest intensity regardless of the wavelength measured. The quantification of the cholesterol for the permeation studies was performed as described in [30].

The chromatographic analysis to identify the compounds of the extracts was carried out by liquid chromatography-high resolution tandem mass spectrometry (LC-HRMS/MS) using an Elute OLE UHPLC system interfaced with a quadrupole time-of-flight (QqToF) Impact II mass spectrometer equipped with an electrospray source (ESI) (Bruker DaltoniK GmbH, Bremen, German). Chromatography separation was carried out on an Intensity Solo 2 1.8 µm C18 100 × 2.1 mm column (Bruker Daltonics, Bremen, Germany). The mobile phase, the elution conditions, and data acquisition by mass spectrometry were done as described in [31]. The column and the sampler were maintained at 35 °C and 10 °C, respectively. The mass analysis was carried out in ESI negative and positive mode, the optimized parameters being: −3.5 kV and +4.0 kV; end plate offset, 500 V, nebulizer gas (N_2_) 2.0 bars; dry gas (N_2_), 8 Lmin-1; dry heater, 200 °C; collision cell energy was set to 5.0 eV. The internal calibration was performed with 250 mL H_2_O, 50 mL iPrOH, 750 µL acetic acid, 250 µL formic acid, and 0.5 mL 1N NaOH solution on HPC mode. The acquired data were processed by DataAnalysis 4.1 software (Bruker Daltonik GmbH, Bremen, Germany). This identification was carried out by taking into account the suggestions from the DataAnalysis^®^ program version 4.4 from BRUKER and the Metlin database. Putative structures for the identified compounds were drawn by MassFrag from Bruker (Bremen, Germany) taking into account the MS/MS fragmentation.

### 2.6. Total Phenol Content Quantification

Total phenolic compound content was determined spectrophotometrically as described in [32]. The absorbance was measured at 760 nm in triplicates. The standard curve (y = 0.007 x + 0.0061; *R*^2^ = 0.9958) was performed with serial phloroglucinol solutions (0.2–100 ug/mL). The concentration of the total phenolic compounds was calculated as micrograms of phloroglucinol equivalents (PGE) per milligrams of dry extract as the mean of three replicates.

### 2.7. Determination of Antioxidant Activity

Antioxidant activity was measured by 2,2-Diphenyl-1-picrylhydrazyl (DPPH) method as described in [30]. To calculate the percentage of antioxidant activity, the following expression was used: AA (%) = [(A control − A sample)/A control] × 100

In which AA (%) corresponds to the percentage of antioxidant activity, A control refers to the absorbance of the control sample containing water, and A sample to the absorbance of the different extract solution.

### 2.8. Acetylcholinesterase Activity

Acetylcholinesterase enzymatic activity was measured using the method described in [30].

### 2.9. HMG-CoA Reductase Activity

The inhibition of the enzymatic activity of HMGR was determined by measuring the decrease in NADPH (Reduced for of Nicotinamide Adenine Dinucleotide Phosphate) absorbance in the presence of HMG-CoA substrate using the HMG-CoA reductase assay kit (Sigma-Aldrich, Barcelona, Spain) following the manufacturer’s indications and the process described in [32].

### 2.10. Permeation Studies

Caco-2 cells (ATCC#HTB37), a human colorectal adenocarcinoma epithelial cell line, were cultured in RPMI medium supplemented with 10% FBS, 100 U/mL penicillin, 100 U/mL streptomycin, and 2 mM L-glutamine, in T25 cell culture flasks at 37 °C in an atmosphere with 5% CO_2_. The culture medium was changed every 48–72 h. The transport and metabolism experiments and the calculations were performed as described in [33]. For these studies, 0.25 mg/mL of ocean sample extract, the methanol fraction containing the phlorotannins purified by Solid Phase Extraction (SPE), and 5 mM of cholesterol were used.

### 2.11. SEM Observations

The microstructure of the different extracts was investigated by SEM as described in [34].

### 2.12. Statistical Analysis

The results were expressed as average ± standard deviation and the analysis of variance (ANOVA) was performed with *p* = 0.05, using the software Microsoft^®^ Excel 2016 (Microsoft Office 365).

## 3. Results

### 3.1. Chromatographic Characterization and Phenolic Content Determination According to Several Origins and Different Extraction Processes

To evaluate the biological activity of *F. vesiculosus,* that can be used as food in soups or salads or acquired in capsules as food supplement, extracts obtained from *Fucus* of different origins and prepared by several extraction methods were studied. The comparison between all the methods of extraction and sources of algae was carried out analyzing the chromatographic profile of the compounds present in the different extracts before and after SPE purification and determining their total phenolic content. Since algae composition is affected by environmental factors, different sources of *F. vesiculosus* were studied. Beyond the different sources of *F. vesiculosus*, studies were performed using several conditions of aqueous extractions: extraction with water at 25 °C (room temperature) for 24 h and extraction with water at 100 °C for 30 min to simulate a boiling cooking process, followed by a SPE purification. The samples from the ocean were bought already dry, samples from the Tagus were dried by lyophilization and in the oven at 180 °C, and the capsules consisting mainly of midrib part of the algae were already dry.

#### 3.1.1. Phenolic Profile Characterization by RP-HPLC-DAD

**Figure 1:**
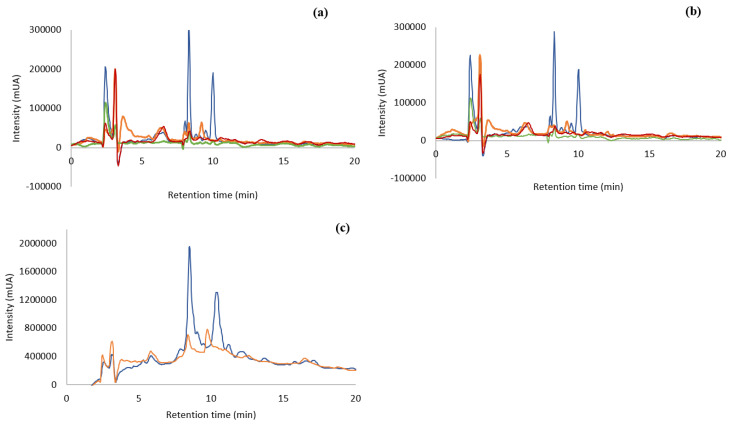
RP-HPLC-DAD of aqueous extract (100 µg/mL) from *F. vesiculosus* (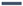) Ocean samples; (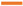) Tagus samples; (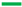) capsules; (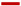) Tagus samples oven-dried. (**a**): Ocean, Tagus, capsules, and Tagus oven-dried extracted at 100 °C, 30 min; (**b**): ocean, Tagus, capsules, and Tagus oven-dried extracted at 25 °C, 24 h; (**c**): ocean and Tagus samples extracted at 100 °C and purified by SPE.

The different extracts were analyzed by Reverse Phase-High Performance Liquid Chromatography – Diode Array Detector (RP-HPLC-DAD). Figure 1 shows the chromatograms of the different extracts, all injected in the concentration of 10 mg/mL. As observed from Figure 1a, *F. vesiculosus* extracted with water at 100 °C gave different chromatographic profiles according to the alga origin. The chromatogram of ocean sample presented nine peaks, the Tagus sample—eight, the Tagus oven-dried—six, and the capsules only presented five peaks. The extract of the ocean samples was the one with higher number and higher intensity of compounds, mainly the peaks with retention time of 8 and 10 min.

The capsules (Figure 1a,b) have almost no phenolic compounds or other type of biological material that can absorb under the Ultraviolet-Visible (UV-Vis) – light.

The chromatographic profile of the different algae extracts with water at room temperature (Figure 1b) is very similar to the results of the extraction with hot water (Figure 1a). In both situations the ocean sample showed the highest number of compounds (9) together with the highest intensities. Comparing both temperatures of extraction for the ocean samples, the peaks at 8 and 10 min have higher intensities, (268001 mUA and 170,626 mUA at 100 °C and 252,534 mUA and 162,551 mUA at 25 °C, respectively) that is an increase of 6 and 5% of area, respectively, when the extraction is carried out with boiling water. Therefore, each temperature of extraction leads to similar profiles, but the extraction at 100 °C produces higher intensities of compounds.

#### 3.1.2. Separation of Bioactive Metabolites by Solid Phase Extraction (SPE)

The chromatographic analyses indicated that there were a few compounds in the extracts that were able to be absorbed in the UV-VIS region. Compounds, such as polysaccharides, that have high solubility in water as well as other bioactive compounds co-extracted with phlorotannins, lead to an extract with compounds that are not absorbed in the UV-Vis region. This may be preventing the visualization of phenolic compounds in the chromatographic process. Knowing that phenolic compounds are absorbed in this region of the spectrum, a purification of the matrix extracted with water was accomplished by using SPE methodology, C18 Sep-Pak cartridge (Waters^TM^, Milford, MA, USA) with the ocean and Tagus samples boiled at 100 °C, as their chromatographic profile showed a higher number of compounds than the capsules or the dried-in-the-oven sample. The objective of this purification was to separate the phlorotannins and other bioactive compounds from non-phenolic metabolites [29]. The fraction containing the bioactive molecules was obtained in the methanol elution step. This process was carried out with the Tagus samples and the ocean samples, extracted at 100 °C, since these extracts presented higher concentration of compounds. The chromatographic analysis carried out by RP-HPLC-DAD is shown in Figure 1c. This purification process produced an increase in the compounds with retention time 8 min of approx. 9 and 11 times, for the ocean and Tagus extract respectively, while the increase in compounds with retention time 10 min was of approx. 9 times, for both the ocean and Tagus extract. This indicated an increase in the compounds absorbing in the UV-Vis region with the SPE purification.

#### 3.1.3. Total Phenolic Content

**Figure 2:**
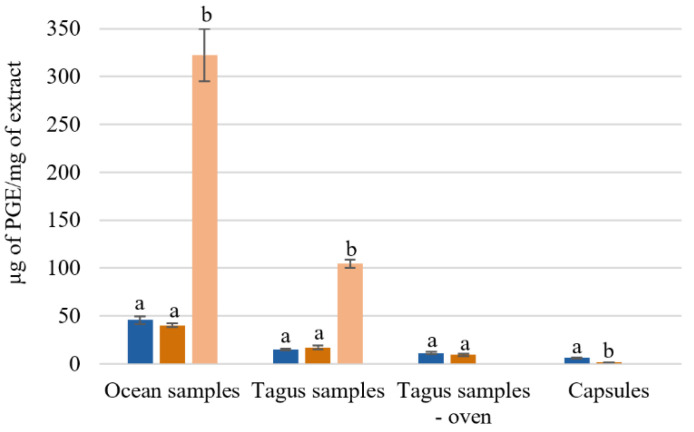
Total phenol content of *Fucus vesiculosus* extracts obtained by different aqueous extraction processes. Total phenolic content (µg of PGE/mg of extract); different superscript letters (**a**,**b**) correspond to values in the same extraction condition that can be considered statistically different (*p* ≤ 0.05). (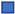) H_2_O 25 °C; (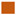) H_2_O 100 °C; (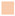) SPE purification (H_2_O 100 °C).

The effect of the extraction temperature on the total phenol content of ocean samples, Tagus samples, and capsules can be observed in Figure 2. One possible explanation for the different amount of total phenol content is the geographic location where the algae were collected, as all the procedures were identical for both ocean and Tagus samples.

The aqueous extracts from the ocean samples presented a higher concentration of µg of phloroglucinol equivalents (PGE)/mg of extract. Comparing with the Tagus samples, the ocean samples had 2–3 times more phloroglucinol equivalents, 45.6 ± 3.8 and 40.2 ± 2.1 µg PGE/mg Ext, while the Tagus samples present 15.0 ± 1.2 and 17.1 ± 2.1 µg PGE/mg Ext, with extraction at 25 °C, and 100 °C, respectively (Figure 2). For the ocean samples, with the SPE purification, the total phenol content increased from 48.8 ± 2.4 to 322.39 ug PGE equivalents/mg of extract. While for the Tagus samples it increased from 21.8 ± 2.5 to 104.7 ± 4.1 μg PGE equivalents/mg of extract (Figure 2).

The results showed that the process of drying in oven leads, approximately, to losses of 25–45% on the total phenolic content 11.1 ± 1.4 and 9.2 ± 1.2 µg PGE/mg Ext with extraction at 25 °C and 100 °C, respectively. The capsules *F. vesiculosus* have lower concentration of PGE equivalents 6.1 ± 0.7 and 0.7 ± 0.3 µg PGE/mg Ext with extraction at 25 °C, and 100 °C, respectively (Figure 2). These results agree with the expected, as the capsules, used as a food supplement, are essentially constituted by fibers.

#### 3.1.4. SEM Observations of *F. vesiculosus* Particles Obtained by Different Drying Methods and Extraction Processes

**Figure 3:**
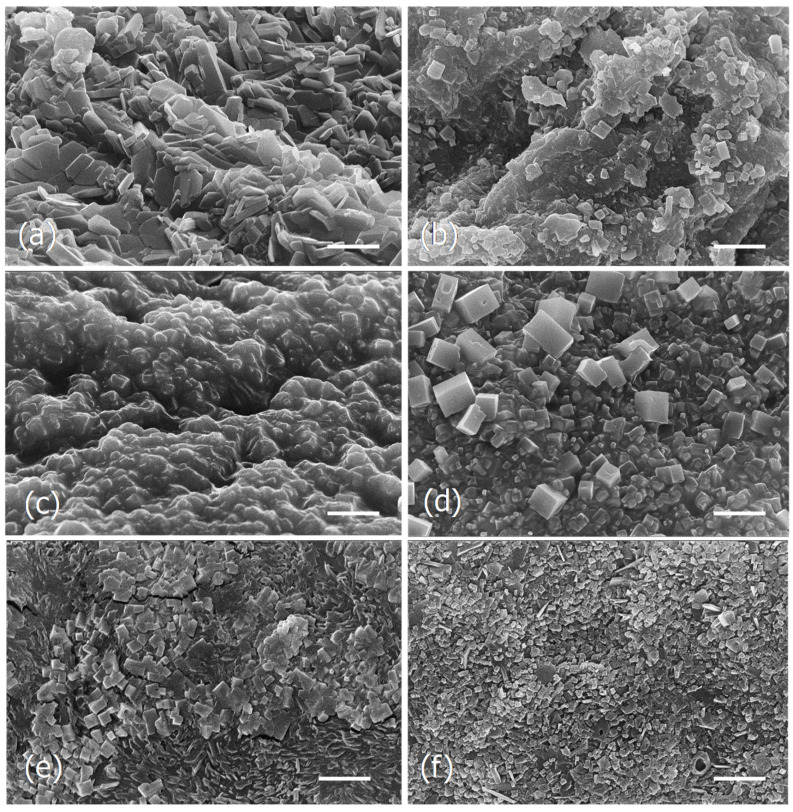
Scanning electron micrographs of the dried particles of Tagus algae obtained by different drying and extraction processes. (**a**), Lyophilized algae. (**b**), Dried in the oven (180 °C, 30 min). (**c**), Lyophilized and extracted at 25 °C. (**d**), Dried in the oven and extracted at 25 °C. (**e**), Lyophilized and extracted at 100 °C. (**f**), Dried in the oven and extracted at 100 °C. Scale bars = 5 μm.

**Figure 4:**
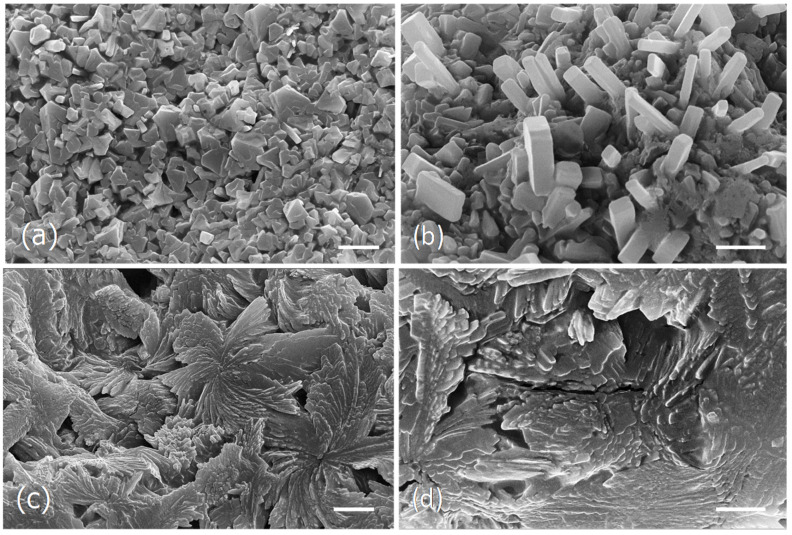
Scanning electron micrographs of the dried particles from the ocean algae and capsules extracts obtained at 100 °C. (**a**,**b**), Ocean samples extract microstructure. (**c**,**d**), Capsules extract microstructure. Scale bars = 10 μm (**a**,**c**); 5 μm (**b**,**d**).

In an attempt to see if the different drying and extraction processes could be reflected into different microscopic changes, SEM analysis was carried out. The analysis was performed with the Tagus samples dried by lyophilization or in the oven before and after the various water extraction processes (Figure 3) and with the aqueous extracts of the ocean algae and capsules obtained at 100 °C (Figure 4).

The observations show that the microstructure of the two samples of dried algae, lyophilized, and dried in the oven, were different (Figure 3a,b), as well as the respective extracts obtained at 25 °C (Figure 3c,d) and 100 °C (Figure 3e,f). In both cases, the extracts of the algae lyophilized or dried in the oven exhibit groups of cubic and parallelepiped-shaped crystals, but in the aqueous extracts of the lyophilized algae they seem to be embedded in an amorphous matrix (Figure 3c,d).

The water extract obtained from the ocean samples at 100 °C, seem to be essentially constituted by large aggregates of cubic and parallelepiped-shaped crystals of diverse sizes (Figure 4a,b), while the capsules extract obtained at the same conditions appeared as a fibrous framework formed by rosette aggregates made by irregular long-shaped particles of different sizes (Figure 4c,d).

In the current study it was observed that the different drying processes as well as the several extraction methods, although giving identical brownish powder, showed different microstructures.

### 3.2. Biological Activities

Biological activities of the extracts obtained at 100 °C without and with SPE purification were studied by determining its antioxidant activity, measured as scavenging radical capacity, acetylcholinesterase inhibitory activity and the capacity for reducing the cholesterol biosynthesis, as well as the inhibition of cholesterol intestinal permeation. The antioxidant activity was also evaluated for the two temperatures of extraction used.

#### 3.2.1. Free Radical Scavenging Capacity

The antioxidant activity for the different extracts studied was determined using the DPPH scavenging activity. Antioxidant activity reflects the ability to eliminate free radicals that have the capacity to damage cellular constituents, such as proteins, DNA (Deoxyribonucleic acid), and lipids [34]. In this study all extracts were used at a concentration of 0.1 mg/mL.

**Figure 5:**
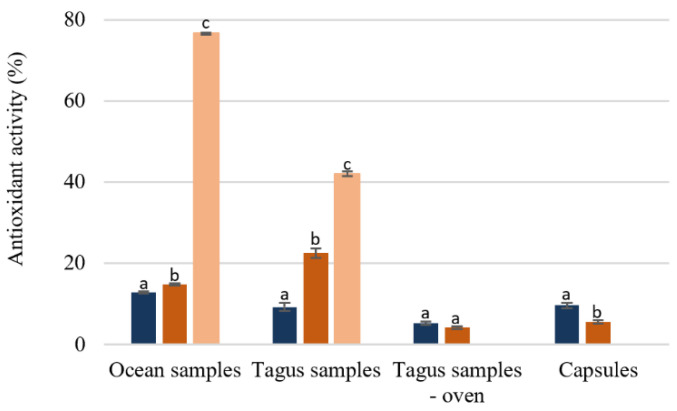
Percentage of antioxidant activity of *F. vesiculosus* extracts (100 µg/mL of extract). Different superscript letters (**a**–**c**) correspond to values in the same extraction condition that can be considered statistically different (*p* ≤ 0.05). (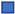) H_2_O 25 °C; (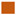) H_2_O 100 °C; (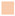) SPE purification (H_2_O 100 °C).

Analyzing the effect of the temperature extraction on the antioxidant activity (Figure 4), the extraction at 100 °C leads to a higher activity, except for the capsules. The ocean samples extracted at 100 °C and purified by SPE was the extract with a higher antioxidant activity (76.54% ± 0.17) followed by the Tagus samples extracted with the same conditions (41.98% ± 0.55). The results present in Figure 4 demonstrate that although the antioxidant activity even in the best situation is lower than the standard like butylated hydroxytoluene (BHT), whose IC_50_ is 15.7 ug/mL [35,36], it would be approximately 60 ug/mL, value similar to those obtained in infusions from medicinal plants [30]. Capsules, as well as the Tagus samples dried in the oven showed a very low antioxidant activity, Figure 5.

#### 3.2.2. Acetylcholinesterase Activity

In this study, only the extracts with higher antioxidant activity were included, but the comparisons were carried out without the purification of the extracts by SPE. The results obtained, summarized in Table 1, showed that the AChE inhibitory activity at 1 mg/mL of the ocean samples was approximately 20 times higher than that presented by the Tagus samples. After the SPE purification the capacity to inhibit the AChE was higher for both extracts compared to the initial aqueous extracts under study. The purified ocean samples extract presented an IC_50_ of 14.97 µg/mL, while the IC_50_ of the purified Tagus extract is almost 56 times higher (840.85 µg/mL), meaning lower activity (Table 1). Comparing with other type of aqueous extracts like those obtained from medicinal plants, these aqueous extracts are much more active than most of the extracts containing also phenolic compounds [25,30]. Nevertheless, both extracts are far less potent than the standard galantamine that has an IC_50_ of 0.14 µg/mL [35], which means that when consumed it will not affect strongly the intestinal motility as the drug might do [37].

#### 3.2.3. HMG-CoA Reductase Inhibitory Activity

For the study of the HMGR inhibition, the high activity before and after the SPE purification was again obtained with the ocean samples, Table 1. The aqueous extract of the ocean samples, at 1 mg/mL, has a percentage of inhibition approximately 27 times higher than the Tagus samples. With the purification of SPE, the ability to inhibit HMGR for ocean samples increased 3–4 times, and for Tagus samples it increased 50 times compared to the respective aqueous extracts initially studied, meaning that there are compounds in these aqueous preparations with the capacity to inhibit this enzyme. After the SPE purification, the IC_50_ value for the purified ocean samples extract is approximately 4.16 µg/mL. The IC_50_ of simvastatin (0.198 ± 0.015 µg/mL) is almost 20 times lower compared to the value determined for the alga water extract, that in this case is a mixture of several compounds, while simvastatin is a pure compound used as a drug.

#### 3.2.4. Cholesterol Permeation

Once the ocean samples extract purified by SPE demonstrated a positive effect inhibiting the rate-limiting enzyme of the cholesterol biosynthesis as previously demonstrated, a study to evaluate the effect of this extract on cholesterol permeation through the intestine was performed. The intestinal barrier was simulated using Caco-2 cells, that have the capacity to differentiate into a monolayer of polarized cells with morphological and functional characteristics of enterocytes of the small intestine [33,38]. *F. vesiculosus* is one of the 22 species of seaweed that is considered safe to be consumed as vegetables and condiments under the European directive (EC 258/97) [1], which means that it is not cytotoxic and can be used for the permeation study.

The ocean sample extract SPE purified at 0.25 mg/mL and the cholesterol at 5 mM in HBSS were added to the apical side of the cells and a control was prepared only with cholesterol in the same concentration. After 6 h into contact with the cells, the cholesterol on the basolateral compartment and inside the cells was quantified by HPLC-DAD. Comparing the permeation of the cholesterol in the presence of the extract with the cholesterol permeation in the control, a reduction of 45.3 ± 4.4% in the cholesterol permeation in the presence of the extract was observed, Table 1.

### 3.3. Compound Identification by LC-HRMS/MS

Based on the better values of the biological activity of the extracts after the SPE purification, a tentative identification of the compounds in the extracts obtained from the ocean and Tagus samples was carried out by HRMS using LC-HRMS/MS. The mass was acquired in positive and negative mode, but only the negative mode is shown here, because identical chromatograms were obtained, the negative mode being the one showing the higher intensity of the compounds. The chromatograms for the ocean and Tagus samples are shown in Supplementary Material Figure S1. The mass analysis and the intensity of the several compounds in the HRMS chromatograms (Figure S1) allowed the establishment of the heatmap shown in Table 2. The aqueous extract from the ocean samples had a higher number of compounds (108 compounds) than the extract of the Tagus samples (85 compounds). On the other side, this last extract had compounds with retention time at 3.2, 6.9, and 9.8 min, compounds 39, 40, and 43 (Table 2) in a much higher intensity than the extract of the ocean samples.

A comparison of the compounds present in both algae extracts was carried out by analyzing the intensities of each peak in a heatmap, Table 2. There are 65 common compounds in both extracts, but to explain the differences in these extracts, the activities of the compounds present only in the ocean sample (compounds 5, 8, 10, 15, 18, 20–27, 29–38, 41, and 44) were identified first, Table 3.

There are compounds in both alga extract with high intensity (compounds 1, 2, 3, 4, 6, 7, 9, 11–14, 16, 17, 19, 28, Table 2) as well as compounds present only in Tagus sample with high intensity, (compounds 39, 40, 42, Table 2). These compounds were also identified, Table 4.

The compounds identified indicated that there are two main groups of chemical structures, phlorotannin derivatives and small peptides, these together represent 94% of the intensities detected. The three compounds that do not belong to this type, compounds 18, 30, and 44 represent only 6% of the intensities determined. Of the identified compounds, 38% were phlorotannins and 50% peptide derivatives. The bioactivities found result from the activities of this mixture. The algae from the ocean has a higher amount of phlorotannins and peptide derivatives than the algae from the Tagus. 14 phlorotannins and 13 peptide derivatives were identified in the ocean sample that were not present in Tagus, while in the Tagus sample only 4 phlorotannins and 3 peptide derivatives were identified. The compounds found in higher amount in the Tagus sample, a glycosidic derivative, an iridoid, and the third one a chroman derivative did not show a strong bioactivity. The phlorotannins detected seem to be phloroglucinol units binding together originating compounds with different degrees of polymerization with molecular weight ranging from 374 Da to 876 Da. This type of phlorotannins are usually called fuhalols and have been detected in other brown alga [39]. The phloroglucinol with higher molecular weight, 930 Da was binding an amino acid residue. A search on the web did not provide description of this type of compound before in algae. On what concerns the small peptides detected in the aqueous extract was composed of 8 amino acid residues, all the others had 4 binding amino acid residues, forming small polymers of mass oscillating between 356 Da and 531 Da. The octapeptide had a molecular weight of 712 Da. In different species of brown alga other small peptides were detected, some with 3 amino acid residues, others with 4, and most of them with higher amino acid sequences [40]. In most of the small peptides detected in *F. vesiculosus* extract essential amino acids are present, indicating the high nutritional value of this food preparation.

## 4. Discussion

In the present study the aqueous extraction at high temperatures leads to higher total phenol content and consequently higher biological activities. These results are in accordance with previous studies that reported that the extraction of phenolic compounds at high temperatures leads to high yields of extraction, once the molecular movement and the solubility increase, facilitating the dissolution of flavonoids from the plant cells and disrupting the cell membranes, facilitating the extraction process [41,42]. The results demonstrated that the process of drying with high temperature also affect the composition of the algae. The Tagus extract dried in the oven presented lower content of phenolic compounds, even when extracted with water at 100 °C, and consequently loss of compounds that were absorb in the UV-VIS light compared with the Tagus extract dried in the freeze dryer. The results show that *F. vesiculosus* from the Atlantic Ocean is different from that collected in the Tagus River estuary, on what concerns the bioactive compounds and, consequently, in their biological activities. The SEM analysis also gave an indication of the different texture in the microstructure of all the extracts.

Independently of genetic characteristics, one possible explanation for the differences between the ocean and the Tagus samples is the fact that in the ocean the algae are exposed to more stressful conditions, such as high salinity, big wave action, and great shear stress, which lead to the synthesis of compounds to stress protection. The analysis by LC-HRMS/MS of the *F. vesiculosus* extracts confirmed that the ocean samples have higher amount of phlorotannins and peptides derivatives comparing with the Tagus samples. Of the 14 phlorotannins identified in the ocean sample, only 3 are also found in the Tagus sample. In relation to the peptide derivatives 11 were identified in the ocean sample but only 1 was also found in the Tagus sample, when analyzed the compounds present in both samples.

To study the enzymatic activity of the different extracts, the acetylcholinesterase (AChE) was chosen as a model enzyme, once AChE inhibitors are used in the treatment of important diseases like Alzheimer’s disease and severe gastrointestinal disorders [30,35]. The search for natural compounds with cholinesterase inhibitory activity arises since synthetic cholinesterase inhibitors have adverse side effects [26,43]. Different natural compounds, including phenolic compounds, have been shown to be potent AChE inhibitors [44]. The results for the inhibition of the acetylcholinesterase can also be explained by the composition of the ocean samples, presenting a higher amount of phenolic compounds like phlorotannins compared to the Tagus algae and higher amount of small peptides. Previous studies with marine algae reported that purified phlorotannins from *Ecklonia stolonifera* algae can prevent the biding of the substrates like acetylcholine (Ach) and butyrylthiocholine to cholinesterase in a non-competitive manner [26]. The different peptide derivatives with phenolic groups identified in the ocean sample can also be inhibitors of the AChE enzyme. Previous studies reported marine-derived peptides as acetylcholinesterase-inhibitors [45,46]. Phlorotannins have in their molecular structure a phloroglucinol moiety, containing an aromatic ring. Compounds containing aromatic rings can show affinity to the enzyme active site because of the presence of Phe residue at the entrance of the enzyme active site [27,35]. Phlorotannins and the small peptides detected in the aqueous extracts can act as AChE inhibitors. Some of the small peptides detected also contain benzene ring in the amino acid residues. These aromatic rings may establish pi-pi interactions with the amino acid at the entrance of the active site pocket [35]. In the present study the value obtained for the inhibition of acetylcholinesterase indicates that the algae, when consumed in a meal, like a soup, may accelerate the digestive tract, also avoiding for instance the cholesterol absorption. This can be an indirect way of reducing the absorption of cholesterol from the diet. It is known that the polysaccharides present in algae have the effect of sequestering cholesterol and diminishing its absorption [12]. With the presence of other bioactive molecules like phlorotannins this effect may be reinforced. In the aqueous extract of the ocean samples there were phlorotannin with high molecular weight. It can be speculated that these compounds have difficulty in permeating the intestinal cell membranes, probably because smaller compounds can penetrate the cell membrane and be active inside the cell. The bioactivities described may be attributed to the small weight molecules of phlorotannins and small peptides. The peptides found are mostly small peptides, with molecular weight between 204 and 740 Da that may permeate the intestinal barrier or may be hydrolyzed during the digestive process.

Hypercholesterolemic levels are the main causes for the cardiovascular diseases, the leading causes of death worldwide [28,47]. The drugs widely used to reduce the cholesterol levels act by inhibiting the HMGR or by preventing its absorption though the intestinal barrier [48]. HMGR regulates the de novo biosynthesis of the intracellular cholesterol, being the most common HMGR inhibitors those that include lovastatin, fluvastatin, simvastatin, pravastatin, pitavastatin, rosuvastatin, and atorvastatin. Although statins are well-known for the effective cholesterol-lowering therapy, they can cause adverse effects, which leads to the search for natural drugs [49]. The in vitro studies here presented demonstrated that the aqueous ocean extract of *F. vesiculosus* rich in phlorotannins and peptide derivatives have the capacity to decrease the synthesis of cholesterol by inhibiting the HMGR. Although the active site of HMGR has hydrophobic amino acid residues with which the usual enzyme inhibitors, statins, can interact, it also has polar residues with which compounds having phenolic structure can bind [50]. Phlorotannins and the small peptides have more hydrophilic groups able to establish hydrogen bonds that can attach to these hydrophilic amino acid residues in the MHGR active site.

This extract also has the potential to decrease the permeation of the cholesterol through a simulated intestinal barrier, with a similar value to those obtained with medicinal plants aqueous extracts containing phenolic compounds [33,34,51]. Cholesterol permeation may be affected because of the competition of bioactive compounds for membrane transporters like NPC1L1, which transport cholesterol into the intestinal cell or by increasing the efflux from inside the cells, through the ABCG5 and ABCG8 transporters [48]. Similar studies, with plants have already demonstrated that compounds such as phenols and flavonoid derivatives can interfere with the cholesterol membrane transport proteins by these types of mechanisms [52,53]. The results here reported are also in agreement with previous in vivo studies that demonstrated that marine-derived peptides have the capacity to lower the level of plasma and hepatic cholesterol [2] and phlorotannins like eckol and dieckol have a hypolipidemic effect [20,47]. However, nothing was reported about phlorotannins with fuhalol structure as those referred to in the present work.

In all the studies done in the present work the ocean sample demonstrated better activity relatively to the Tagus sample. This can be described to the presence of a higher number of secondary metabolites, including small peptides and phlorotannins. In fact, there was a correlation, in the present study, between the total phenolic content and the higher activities detected.

The results here presented also indicated that the way of preparing a meal from algae is very important. If the algae are dried in the oven before the meal preparation much of the bioactivity will be lost. Soups seem to be a good way of preparing the algae, as it corresponds to the extract obtained at 100 °C that showed the higher values for the different in vitro activities studied. Here, we provide valuable insights on the effects of consuming *F. vesiculosus* algae depending on the way of cooking and on the origin of the algae. This study can be an opening door for future development of an active ingredient, seaweed, derived for pharmaceutical and food industries.

## 5. Conclusions

When required to collect algae to prepare food with high bioactivity it is better to select the ocean than the river estuary. To prepare the algae, drying in the oven should not be used; it is advisable to dry through lyophilization and prepare the soup. The alga can be washed without losing many of its bioactive compounds. This can lead to a meal containing phlorotannins and small peptides with the capacity to inhibit AChE and reduce diet cholesterol intestinal permeation as well as hypercholesterolemia through the inhibition of HMGR.

## Figures and Tables

**Table 1:** Enzyme inhibitory activity of acetylcholinesterase and HMGR of the ocean and Tagus samples aqueous extracts (100 °C, 30 min) and after SPE purified extract.

Extract	Acetylcholinesterase Inhibition		HMGR * Inhibition		Inhibition of Cholesterol Permeation (%)
	1000 µg/mL	IC_50_ (µg/mL)	10 µg/mL	IC_50_ (µg/mL)	
Ocean samples	89.96% ± 0.54 ^a^	210.86 ± 22.11 ^a^	27.74% ± 1.60 ^a^	-	-
Ocean samples–SPE fraction	-	14.97 ± 0.13 ^b^	86.41% ± 3.20 ^b^	4.16 ± 0.11	45.3 ± 4.4
Tagus samples	4.199% ± 0.36 ^b^	-	1.07% ± 0.65 ^c^	-	-
Tagus samples–SPE fraction	60.44% ± 1.21^c^	849.80 ± 10.24 ^c^	52.95% ± 3.17 ^d^	-	-

* 3-hidroxi-3-methyl-glutaril-CoA reductase; Superscript letters ^(**a**–**d**)^ correspond to values in the same column that can be considered statistically different (*p* = 0.05).

**Table 2:** Heatmap representing the intensity of all compounds present in the Tagus and ocean samples after SPE purification. The green values correspond to the compounds with proposed identification and respective compound number. Intensity: 160000; 1000.

	Mass [M − H]^−^	Samples Origin			Mass [M − H]^−^	Samples Origin			Mass [M − H]^−^	Samples Origin	
N^o^	*m/z*	Ocean	Tagus	N^o^	*m/z*	Ocean	Tagus	N^o^	*m/z*	Ocean	Tagus
	**1.0–1.3 min**			**1.9–2.0 min**					268.9734		
	102.9572				191.0198				516.9721		
1	181.0722				117.0194				268.9734		
	146.0460				269.0874				494.9909		
	165.0406				203.9732				531.0673		
	227.0773				223.0820				536.1138		
	240.0080				230.9893				699.1822		
2	191.0201			7	276.0184			**3.2–3.4 min**			
	217.0484				384.1527				164.0715		
	219.0457			8	455.1898				185.0454		
	126.9051				713.2366				232.0592		
	111.0091			9	497.0728			16	277.0932		
	**1.5–1.6 min**			10	875.2911				299.0754		
	117.0193				**2.0–2.1 min**			17	351.1334		
	140.0115			11	327.1299				375.0613		
	149.9627				425.0990				397.0428		
	181.0715			**2.1–2.3 min**				18	410.1695		
	219.9681			12	237.0078			19	555.1934		
3	256.0940				475.0231				855.2730		
	300.9335				265.9819			**3.5–3.6 min**			
	319.9066			13	125.0247			20	355.1642		
	322.9155				174.9560			21	497.0748		
	338.8896			**2.4–2.6 min**					387.0309		
	370.1380			14	246.9916			**4.5–4.7 min**			
4	403.0557				328.9980			22	355.1622		
5	445.0962			15	373.0572				227.1037		
	541.1551				680.2201				453.1301		
6	551.1836				536.1125			23	711.3323		
					248.9894						
	**4.7–4.8 min**				**6.1–6.2 min**				**12.7–12.8 min**		
24	203.0824				131.0714				174.9558		
25	291.0988				186.1133			43	527.2535		
	369.1787				785.3471						
26	419.1573				**6.7–6.8 min**						
27	621.0891				147.0452						
	**5.0–5.1 min**			40	165.0557						
	138.0562				206.0822						
28	175.0611				119.0504						
	187.9041				331.1190						
	375.8086				353.1007						
29	745.1056				619.2774						
**5.4–5.7 min**				41	930.1220						
30	324.1576				1054.6409						
31	369.1787				**7.8 min**						
	405.1553			42	361.1503						
	427.9317				383.1323						
32	437.1652				587.2592						
33	467.1465				433.1622						
34	739.3642				9.8–10						
	241.1192				394.9648						
35	531.2215				395.9679						
36	682.0892				**10.9–11.1 min**						
37	806.1051				396.9627						
38	869.1216				160.8416						
	467.1461				162.8387						
**5.7–5.8 min**					174.9556						
39	247.0823				447.1349						
	145.0598										
	269.0646										

**Table 3:** Identification proposal of the compounds detected by LC-HRMS/MS in ESI negative mode only present in the ocean samples extract. «No» indicates compound number corresponding to Table 2.

Rt (min)	[M − H]^−^ *m/z*	Molecular Formula	Error (ppm)	Fragmentation (%)	Name	N^o^
1.5	445.0962	C_18_H_22_O_13_	3.3	314 (50%); 221 (100%); 80 (60%)	Phloroglucinol derivative	5
1.9	455.1898	C_18_H_28_N_6_O_8_	−0.5	238.0839 (96.97%); 126.0196 (37.10%); 88.0404 (23.56%)	Peptide derivative ThrGluHisAla	8
1.9	875.2911	C_49_H_48_O_15_	−0.2	829 (100%); 125 (7%); 179 (14%); 383 (16%)	Phloroglucinol derivative (7 units)	10
2.6	373.0572	C_18_H_14_O_9_	−1.5	355 (37%); 141.0192 (100%); 207.0303 (20%)	Phloroglucinol derivative (3 units)	15
3.2	410.1695	C_17_H_25_N_5_O_7_	−4.8	136 (45%); 109 (45%); 366 (58%); 339 (35%)	Benarthin	18
3.5	355.1642	C_15_H_24_N_4_O_6_	−4.5	311 (74%); 252 (19%); 194 (24%); 96 (46%)	Tetrapeptide ProProGlySer	20
3.6	497.0748	C_24_H_18_O_12_	0.6	125 (45%); 141 (30%); 372 (20%); 356 (20%)	Phloroglucinol derivative (4 units)	21
4.5	355.1622	C_15_H_24_N_4_O_6_	0.2	311 (12%); 238 (95%); 240 (82%); 183 (3 7%); 116 (100%); 70 (8%)	Tetrapeptide ProSerGlyPro	22
4.5	711.3323	C_30_H_48_N_8_O_12_	−2.2	355 (100%); 129 (2%); 116 (9%); 356 (20%)	Octapeptide of ProProGlySer	23
4.7	203.0824	C_11_H_12_N_2_O_2_	1	116.0508 (100%); 74.0251 (37,77%)	Tryptophan	24
4.7	291.0982	C_14_H_16_N_2_O_5_	1.5	Didn’t fragment	Tryptophan derivative	25
4.7	419.1573	C_19_H_24_N_4_O_7_	0	Didn’t fragment	Tetrapeptide of (GluSerTrp)	26
4.7	621.0891	C_30_H_22_O_15_	−2.5	125 (84%); 141(6%); 165 (6%); 485 (3%); 289 (3%); 478 (50%)	Phloroglucinol derivative (5 units)	27
5.0	745.1039	C_36_H_26_O_18_	−0.5	125 (29%); 141 (14%); 497 (100%); 413 (4%)	Phloroglucinol derivative (6 units)	29
5.4	324.1576	C_15_H_22_N_3_O_5_	−1.9	209.0944 (45.86%); 166.0868 (34.90%); 114.0565 (100%);	N-(5-Hydrazinyl-5-oxopentyl)-3,4,5-trimethoxybenzamide	30
5.4	369.1787	C_16_H_26_N_4_O_6_	−0.1	325.1879 (19.73%); 238.0834 (87.76%); 130.0872 (100%) 58.0303 (15.04%);	Tetrapeptide ThrProProGly	31
5.4	437.1652	C_19_H_26_N_4_O_8_	3.8	392 (23%); 320 (11%); 348 (1%)	Tetrapeptide GlyAspPheThr	32
5.4	467.1459	C_17_H_32_N_4_O_5_S_3_	−1.7	423; 319 (0.4%)	Tetrapeptide LeuCysCysMet	33
5.4	739.3642	C_32_H_52_N_8_O_12_	−2.9	369 (100%); 143 (1%); 238 (5%)	Peptide derivative	34
5.6	531.2215	C_24_H_32_N_6_O_8_	1.0	Didn’t fragment	Tetrapeptide ValAspTrpAsn	35
5.6	682.0892	C_27_H_25_NO_20_	−4.4	125 (44%); 141 (27%); 134 (13%); 495 (43%)	Phloroglucinol derivative	36
5.6	806.1051	C_33_H_29_NO_23_	−0.8	369 (95%); 195 (21%); 761(23%); 238 (31%); 567(19%)	Phloroglucinol derivative	37
5.6	869.1216	C_42_H_30_O_21_	-2.4	125 (40%); 141 (9%); 137 (9%);194 (6%); 335 (14%);	Phloroglucinol derivative (7 units)	38
6.7	930.1225	C_39_H_32_NO_26_	−0.7	Didn’t fragment	Phloroglucinol and amino acid derivative	41
12.7	527.2535	C_32_H_36_N_2_O_5_	3.1	418 (0.2%); 165 (0.8%); 130 (0.3%)	Chaetoglobosin A	44

**Table 4:** Identification proposal of the compounds detected by LC-HRMS/MS in ESI negative mode present in the ocean and Tagus (river estuary) extracts and present only in the Tagus with high intensity. «No» indicates compound number corresponding to Table 2.

Rt (min)	Accurate [M − H]^−^ *m/z*	Molecular Formula (Error, ppm)	Fragmentation (%)	Proposed Compound	N^o^
1.1	181.0722	C_6_H_14_O_6_ (2.3)	-	Mannitol *	1
1.2	191.0201	C_6_H_8_O_7_ (2.0)	-	Citric acid *	2
1.5	256.0941	C_10_H_16_N_3_O_5_ (5.4)	212.10(6); 194.09(93); 166.06(7); 141.06(9); 82.03(32)	Cytidine derivative	3
1.5	403.0555	C_12_H_20_O_13_ (−3.7)	384.98(2); 370.13(4); 231.45(1) 219.96(21); 149.96(100); 96.96(2); 79.95(46)	Galactose-sulfate derivative	4
1.6	551.1836	C_19_H_36_O_18_ (−5.3)	506.12(10); 341.10(15); 325.11(6); 179.05(100); 161.04(17)	Glycidyl compound	6
1.9	276.0187	C_9_H_12_NO_7_S (−3.0)	230.01(9); 196.06(100); 179.05(45);135.04(92)	Tyrosine sulfate	7
1.9	497.0727	C_24_H_18_O_12_ (−2.6)	479.05(10); 383.17(26); 331.04(27);287.05(6); 165.01(100); 137.02(26)	Phloroglucinol derivative	9
2.0	327.1299	C_12_H_24_O_10_ (−1.3)	238.08(26); 101.07(8); 88.04(100)	Glycosidic derivative	11
2.1	237.0078	C_10_H_6_O_7_ (−4.4)	153.05(12); 136.73(1); 123.04(13); 96.96(93)M 79.95(100)	3. ,5,7-Trihydroxy-4-oxochromene-2-carboxylic acid	12
2.2	125.0247	C_6_H_6_O_3_ (−2.6)	-	Phloroglucinol *	13
2.4	246.9916	C_8_H_8_O_7_S (−0.4)	164.93(2); 121.02(23); 108.02(100); 80.96(76)	Vanillic acid sulfate	14
3.2	277.0930	C_11_H_18_O_8_ (0.7)	141.05(5); 97.06(100); 91.03(10)	Phloroglucinol glycosidic derivative	16
3.2	351.1334	C_15_H_20_N_4_O_6_ (6)	238.12(5); 181.04(3); 164.07(100)	Tetrapeptide Gly Tyr	17
3.2	555.1930	C_22_H_36_O_19_ (0.1)	277.09(100); 185.04(97); 141.05(23)	Phloroglucinol glycosidic derivative	19
5.0	175.0611	C_7_H_12_O_5_ (0.0)	115.04(45); 85.06(100); 59.01(13)	Butanedioic derivate	28
5.7	247.0823	C_10_H_16_O_7_ (0.1)	145.05(40); 127.04(5); 101.06(100);83.05(97)	Glycosidic derivative	39
6.7	165.0557	C_9_H_10_O_3_ (0.1)	147.04(18); 119.05(60); 91.05(5); 73.99(100)	Chroman-2,5-diol	40
7.8	361.1503	C_16_H_26_O_9_ (−3.3)	233.10(13); 145.05(16); 127.04(11); 88.05(100)	Gibboside isomer	42

* dentification confirmed with standard analysis.

## Supplementary Materials

The following are available online at https://www.mdpi.com/2304-8158/9/7/955/s1, Figure S1: Chromatogram LC-HRMS/MS in negative mode of *F. vesiculosus* from ocean (blue) and from Tagus (black).
