# Supplementary material for: Effect of Food Preparations on In Vitro Bioactivities and Chemical Components of Fucus vesiculosus
Source: Foods. 2020 Jul 18;9(7):955. doi: 10.3390/foods9070955 (PMC7404634; doi:10.3390/foods9070955)
Supplement: Supplementary file 1 [file foods-09-00955-s001.pdf]

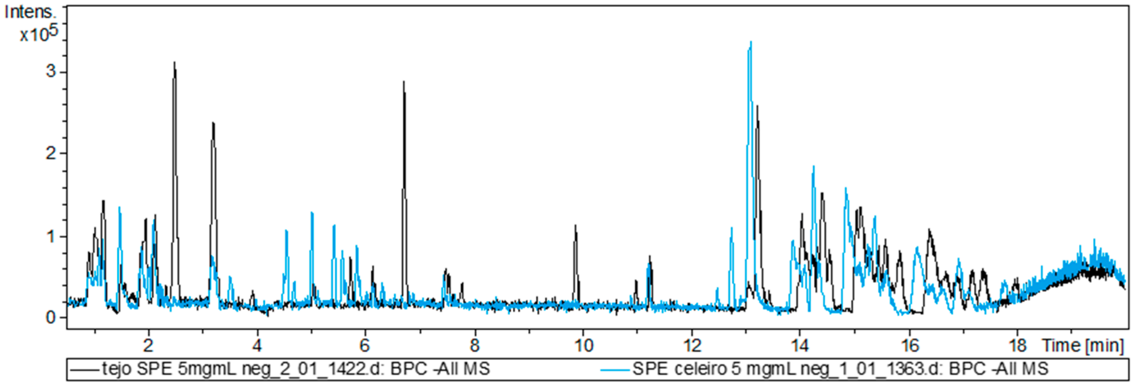

Figure S1. Chromatogram LC-HRMS/MS in negative mode of *F. vesiculosus* from ocean (blue) and from Tagus (black).
